# The Association between *HMGA1* rs146052672 Variant and Type 2 Diabetes: A Transethnic Meta-Analysis

**DOI:** 10.1371/journal.pone.0136077

**Published:** 2015-08-21

**Authors:** Aida Bianco, Eusebio Chiefari, Carmelo G. A. Nobile, Daniela Foti, Maria Pavia, Antonio Brunetti

**Affiliations:** Department of Health Sciences, University “Magna Græcia” of Catanzaro, Catanzaro, Italy; University of Leipzig, GERMANY

## Abstract

The high-mobility group A1 (*HMGA1*) gene has been previously identified as a potential novel candidate gene for susceptibility to insulin resistance and type 2 diabetes (T2D) mellitus. For this reason, several studies have been conducted in recent years examining the association of the *HMGA1* gene variant rs146052672 (also designated IVS5-13insC) with T2D. Because of non-univocal data and non-overlapping results among laboratories, we conducted the current meta-analysis with the aim to yield a more precise and reliable conclusion for this association. Using predetermined inclusion criteria, MEDLINE, PubMed, Web of Science, Scopus, Google Scholar and Embase were searched for all relevant available literature published until November 2014. Two of the authors independently evaluated the quality of the included studies and extracted the data. Values from the single studies were combined to determine the meta-analysis pooled estimates. Heterogeneity and publication bias were also examined. Among the articles reviewed, five studies (for a total of 13,789 cases and 13,460 controls) met the predetermined criteria for inclusion in this meta-analysis. The combined adjusted odds ratio estimates revealed that the rs146052672 variant genotype had an overall statistically significant effect on increasing the risk of development of T2D. As most of the study subjects were Caucasian, further studies are needed to establish whether the association of this variant with an increased risk of T2D is generalizable to other populations. Also, in the light of this result, it would appear to be highly desirable that further in-depth investigations should be undertaken to elucidate the biological significance of the *HMGA1* rs146052672 variant.

## Introduction

Type 2 diabetes (T2D) mellitus is a heterogeneous complex disease in which both predisposing genetic factors and precipitating environmental factors contribute to the development of the disease [1,2]. Because of the pandemic explosion of T2D, along with its high morbidity and mortality, and its effect on health care costs, many studies have been performed in recent years to elucidate the pathogenetic mechanisms of this disease [3]. While the adverse impact of environmental factors (increased caloric intake and sedentary lifestyle) is generally accepted and easy to identify [4], the clinically relevant genes associated with T2D still remain to be elucidated.

So far, over 50 gene variants have been associated with an increased risk of developing T2D. Whereas most of them are involved in pancreatic beta-cell function, which means in insulin secretion defects, some of them have been related to peripheral insulin resistance, which impairs peripheral glucose uptake [5]. However, despite the efforts and the recent genome-wide association studies (GWAS), these genetic variants explain only a small proportion of heritability of T2D [6,7], a phenomenon referred to as the “missing heritability” problem [8], which may result from the involvement of rare variants not included in the GWAS database, or variants having a minor allele frequency below the minimum threshold value (5–10%) of GWAS, or from the action of multiple genes that interact with each other in an epistatic manner [5,6].

In this context, a new rare variant, rs146052672, which consists of a C insertion at position −13 of exon 6 (c.136-14_136-13insC) of the *HMGA1* gene has been recently associated with increased risk of insulin resistance and T2D [9]. The *HMGA1* gene encodes the nuclear architectural factor HMGA1, a non-histone basic protein that binds to AT-rich sequences of DNA via AT hooks, facilitating the assembly and stability of multicomponent enhancer complexes, the so-called ‘‘enhanceosomes”, that drive gene transcription in response to multiple extracellular and intracellular signals [10,11]. The biological plausibility linking HMGA1 to T2D is supported by the findings that HMGA1 is a key element in the transcriptional regulation of genes coding for enzymes and proteins implicated in insulin signaling transduction and glucose metabolism [12–18]. Consistently with these findings, defects in HMGA1 expression and/or function have been previously reported in individuals with insulin resistance and T2D [9,19,20], whereas a type 2-like diabetic subphenotype was observed in the context of a more generalized “HMGA1opathy” induced in mice by targeted disruption of the *HMGA1* gene [19,21].

The *HMGA1* rs146052672 variant was first detected in ~8% of patients with T2D in three separate populations of white, European descent (Italy, US, and France) [9]. Association of this variant with T2D was not replicated in a subsequent study that involved a heterogeneous French population [22]. Later, however, it was reported that the *HMGA1* rs146052672 variant was significantly associated with T2D in a Chinese population study [23], whereas non univocal results were obtained among Hispanic-American populations of the US [24,25]. Furthermore, evidence implicating the rs146052672 variant as one conferring cross-ethnicity risk for the development of insulin-resistance-related conditions has been provided more recently, in a case-control study from Italy and Turkey, in which an increased risk of metabolic syndrome was seen among carriers of this variant [26]. No other studies have investigated the association between *HMGA1* rs146052672 variant and T2D risk, and the conclusions remain controversial rather than conclusive.

Meta-analysis provides a quantitative way to combine the results of different studies on the same research question, and to estimate and explain their diversity. Therefore, conclusions from a meta-analysis are more robust than those from a single study. In addition, meta-analysis is useful to investigate the consistency or heterogeneity of the associations across studies [27]. With this in mind, we carried out the current meta-analysis aimed to investigate the association between *HMGA1* rs146052672 variant and T2D susceptibility.

## Methods

Meta-analysis was undertaken in accordance with guidelines for meta-analysis of observational studies in epidemiology (MOOSE) [28]. Results are reported according to the Preferred Reporting Items for Systematic Reviews and Meta-Analyses (PRISMA) statement [29].

### Search strategy for identification of studies

We sought to identify all studies that investigated the association between *HMGA1* rs146052672 variant and T2D. A comprehensive search of MEDLINE, PubMed, Web of Science, Scopus, Google Scholar and Embase databases was conducted from inception until November 2014. The following keywords in different combinations were used: “type 2 diabetes”, “high-mobility group A1 or HMGA1 or HMGI(Y)”, “variant or polymorphism” as both medical subject heading terms and text words. No language restrictions were applied in the search or study selection. Also, in order to identify additional undetected published studies, we implemented this search by carefully screening the reference list of all retrieved articles, plus the most recent review articles, and the PubMed option “Related Articles”.

### Inclusion and exclusion criteria

Two of the authors independently reviewed all potentially relevant publications to determine whether an article met the inclusion/exclusion criteria. Disagreements were discussed at a team meeting where a final decision was reached. Studies were included in the meta-analysis only if they met all the following criteria: (a) they reported original data from case-control studies performed in the adult population, regardless of the clinical/non-clinical setting where cases and controls were recruited; (b) the outcome was T2D (incident or prevalent); (c) they investigated the association of *HMGA1* rs146052672 variant with T2D, separating results according to ethnic groups; (d) odds-ratio (OR) estimates were reported with 95% confidence intervals (CIs), or sufficient data were available to calculate these estimates; (e) method of genotyping used should have been explained or linked to a reference; (f) studies were published through November 2014. Conversely, studies would be excluded if they were case-only studies or studies of type 1 diabetes, review articles, case reports, letters, comments, editorials and duplicate publications. When the same patient population was reported in several publications, only the most recent or complete study was used. Studies published as abstracts only were excluded. Sample size or the absence of allele frequencies among cases and controls were not exclusion criteria.

### Assessment of study quality

In order to reach an assessment of the quality of each study, two of the authors independently reviewed the studies included in the meta-analysis. All eligible articles were carefully read and scored for quality, and to reduce the possibility of bias, investigators, institutions, country, and journal were blinded for each article. The Newcastle-Ottawa Scale (NOS) [30] was used to assess the methodological quality of included studies. As quite comprehensive and partly validated [30], the NOS, a star-based system allowing a semi-quantitative assessment of nonrandomized study quality, consists of eight items and takes into consideration three major parameters, including selection of participants, comparability of study groups, and exposure. The scale ranges from 0 to 9 stars, with more stars indicating a higher quality study. According to other similar works [31–33], studies that were rated 7 or more stars were considered as high quality studies in the current meta-analysis.

The quality of the included publications was also evaluated according to a scale for methodological quality assessment, specifically designed for genetic studies, which was extracted and modified from previously published studies [34,35]. In this scale, eight items, including the representativeness of cases, sources of controls, ascertainment of T2D and controls, quality control of genotyping examination, sample size, appropriate statistics and adjustment for confounders, and Hardy-Weinberg equilibrium (HWE), were carefully checked. HWE was assessed for each study by using the χ^2^ test or Fisher’s exact test, where appropriate, and only in control groups. P value < 0.05 was considered significant departure from HWE. According to the quality score assessment, a study scoring < 10 was considered a “low-quality” study, whereas a score ≥ 10 represented a “high-quality” study. The lowest score was 0, and the highest score was 15 [36].

The authors discussed their evaluation, and if disagreement did occur, it was solved by re-reading of the text and extensive discussion. To avoid selection bias, no study was excluded based on these quality criteria.

### Data extraction

Data from each included article were independently extracted by two of the authors, using a standardized data collection form. Discrepancies were resolved by discussion and, when necessary, through consultation among the investigators. We attempted to contact the authors of studies if missing data or inconsistencies were detected. The following information was recorded from each study: (a) the first author’s last name, year of publication and country of population studied; (b) study design; (c) definition of disease used in the study; (d) number of participants; (e) any confounding factors for matching or adjustment; (f) method of genotyping used; (g) OR estimates with the corresponding 95% CI for the association between the rs146052672 variant in *HMGA1* gene and T2D risk; (h) type of ethnicity (categorized as Caucasian, Asian, Hispanic, and African).

### Meta-analyses

The primary outcome was the odds of T2D in those with the *HMGA1* rs146052672 variant compared with those without the variant. A dominant genetic model was adopted on the basis of previous observations indicating that: no statistical difference in results was observed between dominant and additive models [23,25]; only one homozygous carrier of the C insertion was identified in the two larger studies including over 17,000 Caucasian individuals [9,22]; no clinical, biological, or biochemical signs of an additive effect were seen in carrier subjects [9, 22–26]. We preferentially used adjusted ORs reported by the authors. If no adjusted estimates were presented, we included the crude estimate. The pooled-effects estimates were used to combine values from the single studies and were expressed as OR and the related 95% CI. OR and CI were obtained using the DerSimonian and Laird random effect model [37], that calculates a weighted average of the ORs by incorporating within-study and between-study variations. The Mantel-Haenszel method (fixed effects model) [38] was also used to evaluate the effect of model assumptions on our conclusions. Compared with the fixed-effect model (that considers only within-study variability), the random-effect model approach generally provides a similar estimate of the OR, but a wider CI if heterogeneity is detected. Statistical heterogeneity was evaluated using the Cochran Q-test, which gives the magnitude of heterogeneity between-studies, and the I^2^ test, which gives the percentage of variation between the study estimates due to heterogeneity rather than chance. An I^2^ between 25 and 50% indicates low heterogeneity, between 50 and 75% moderate heterogeneity and > 75% high heterogeneity [39,40].

### Sensitivity analyses

The studies included in the meta-analysis differed according to several parameters, such as study quality, ethnicity and method of genotyping. Therefore, we performed separate meta-analyses by grouping studies on the basis of quality ratings, group’s ethnicity (for Caucasians and Hispanics), and the use of TaqMan allelic discrimination confirmed by direct-sequencing as a method to detect *HMGA1* mutations. Univariate meta-regressions were also performed to identify the major sources of between-studies variation in the results, using the previously mentioned factors as the possible sources of heterogeneity. Finally, publication bias was examined through the visual inspection of funnel plots and a scattergraph of individual studies effect against a measure of its precision [41], employing the Begg & Mazumdar’s adjusted rank correlation test and the Egger’s regression approach for formal statistical testing [42,43]. All analyses were performed using Stata version 11 software (StataCorp LP).

## Results

### Characteristics of included studies

**Fig 1:**
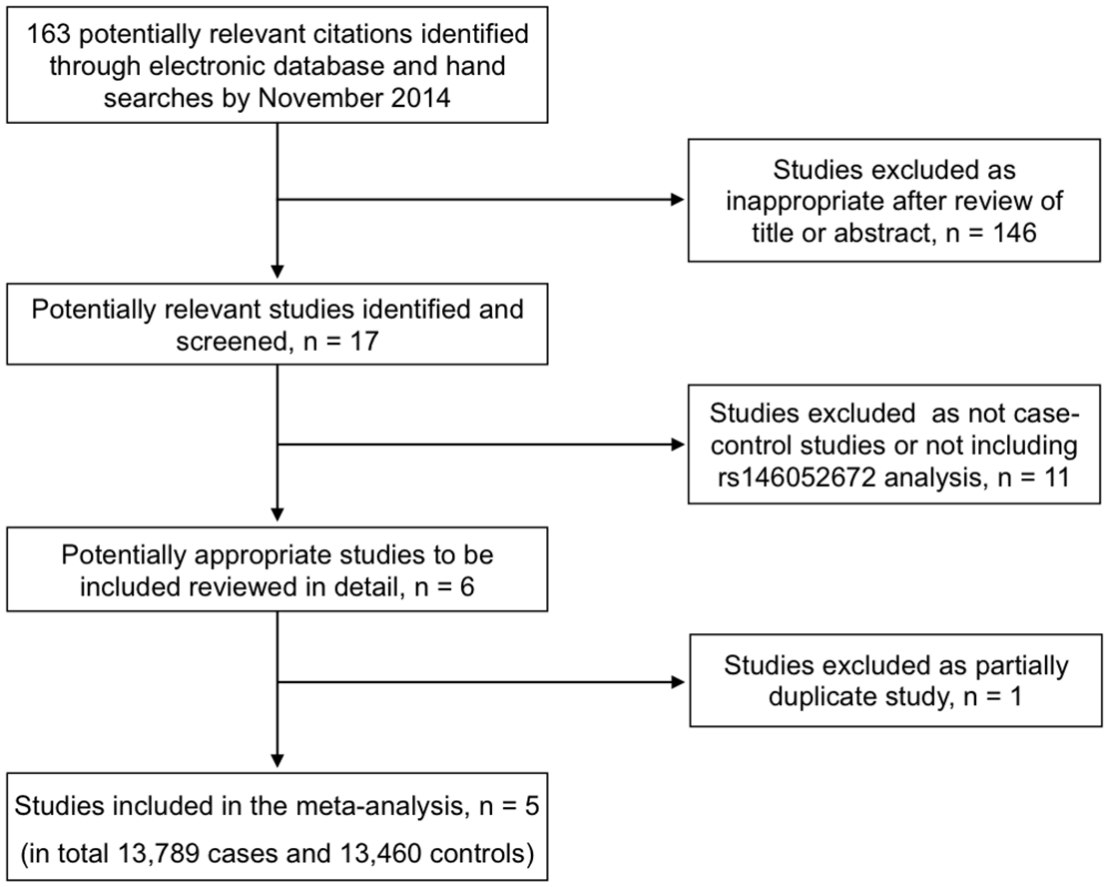
Flow diagram of case-control studies identified in the literature for the association between the *HMGA1* rs146052672 variant and the risk of T2D.

We identified a total of 163 potentially relevant references about HMGA1 and T2D, but on obtaining and reading the articles, only five studies [9,22–25] met the predetermined inclusion criteria for the meta-analysis. Fig 1 shows the flow chart of the literature search results. Among the excluded articles were those in which any one of the following reasons applied: (a) the paper was a review article; (b) the article was duplicative of another publication from the same population; (c) the article was a survey study; (d) insufficient data were provided to determine an estimate of OR and a 95% CI. Reviewers’ agreement on study selection was excellent, as it was higher than 99%. A list of the excluded papers is provided as a Supplementary file (S1 Supporting Information). Summary characteristics of the studies included in the meta-analysis are shown in Table 1. The sample size of the five included case-controls studies varied between 216 and 4,906 for cases and between 153 and 4,387 for controls. The studies were geographically heterogeneous: one study involved European (Italian) and US samples, one study was conducted in France, one in China and two in US. Two not-exposed (control groups) were reported in the Italian population. The largest control group consisted of 2,544 interviewed healthy individuals with neither a personal nor a family history of T2D or related diseases; the smaller control group based on a self-reported medical questionnaire, included 784 healthy individuals screened only for the absence of T2D, without a personal interview. The majority of the study populations was Caucasian (n = 3); one involved Han Chinese population, two Hispanic Americans, and one African Americans. In Hispanic Americans, a much lower minor allele frequency for the *HMGA1* gene variant rs146052672 was observed by Karnes et al. [24], with respect to that reported later by Pullinger et al. [25]. Hispanic Americans include Mexican Americans, Puerto Ricans, Cubans and several other ethnic backgrounds. Genetic diversity in these Hispanic American subgroups has been identified as an important factor explaining the difference in the risk of T2D: higher in Mexican Americans than other US Hispanics [44,45]. Hispanics recruited by Pullinger et al. were predominantly of Mexican origin [25], whereas it is not clear where Hispanic participants were recruited in the other study of Karnes et al. [24]. All studies adjusted for age. Four papers adjusted also for gender and BMI [9,22–24]. Four studies employed the TaqMan allelic discrimination technique and one employed the High Resolution Melting (HRM) method. Genotype distributions in the controls of all included studies were in agreement with HWE. In all five studies, results were reported according to the dominant genetic model, while in only two studies the additive model was also adopted.

**Table 1:** Characteristics of case-control studies on the association of *HMGA1* rs146052672 variant and T2D risk.

Study, [Ref.]	Sub- study	Study area	Race	*N* cases	*N* controls	Case-genotypes wt/wt wt/C C/C	Control-genotypes wt/wt wt/C C/C	HWE controls	Model	Adjustment variables	Adjusted RR/OR estimate (95% CI)	Quality score
Chiefari et al., 2011 [9]	1	Italy	Caucasian	3278	784^a^	3041 237 0	3041 237 0	0.64	D	Age/Gender/ BMI	2.03 (1.51–3.43)	8^c^/11^d^
	2	Italy	Caucasian	3278	2544^b^	3041 237 0	3041 237 0	0.91	D	Age/Gender/ BMI	15.77 (8.57–29.03)	8^c^/12^d^
	3	USA	Caucasian	895	913	895 75 0	895 75 0	0.46	D	Age/Gender/ BMI	1.64 (1.05–2.57)	8^c^/9^d^
Marquez et al., 2012 [22]	–	France	Caucasian	4906	4387	4639 267 0	4639 267 0	0.23	D	Age/Gender/ BMI	1.07 (0.84–1.37)	7^c^/11^d^
Liu et al., 2012 [23]	–	China	Han	2629	2739	2193 422 14	2193 422 14	0.72	D	Age/Gender/ BMI Hypertension Hyperlipidemia Smoking status	1.34 (1.15–1.56)	8^c^/10^d^
								–	A		1.34 (1.16–1.55)	–
Karnes et al., 2013 [24]	1	USA	Caucasian	608	493	NA NA NA	NA NA NA	0.40	D	Age/Gender/ BMI	0.95 (0.44–2.06)	5^c^/8^d^
	2	USA	Hispanic	937	642	NA NA NA	NA NA NA	0.20	D	Age/Gender/ BMI	0.79 (0.49–1.25)	5^c^/8^d^
	3	USA	African	216	153	NA NA NA	NA NA NA	0.10	D	Age/Gender/ BMI	1.51 (0.48–4.74)	5^c^/7^d^
Pullinger et al., 2014 [25]	–	USA	Hispanic	320	805	185 116 19	185 116 19	0.09	D	Age/ Recruitment source	1.44 (1.09–1.90)	7^c^/7^d^
								–	A		1.27 (1.01–1.58)	–

wt, wild-type; C, C insertion; HWE, Hardy-Weinberg equilibrium; D, dominant model; A, additive model.

^a^not interviewed;

^b^interviewed;

^c^according to the NOS [30]

^d^other validated quality scale [34–36].

### Data quality

Based on the NOS, overall quality ratings of the studies ranged from 7 to 8, with a median NOS score of 7 (Table 1). All studies identified cases and controls without knowledge of exposure status, and no known association between control status and exposure. Independent validation of disease diagnosis by fasting glucose level was satisfied in all studies, and the American Diabetes Association standard criteria [46] were used to define the outcome. No studies reported response rate. When assessed with a scale specifically designed for association genetic studies, the estimated quality of all included studies was in the range of 7–12 scores (Table 1). All studies assessed the association between genotypes and T2D with appropriate statistics and adjustment for confounders. Consecutive selection of case population with clearly defined sampling frame and controls drawn from the same sampling frame as cases, were satisfied in two and three studies, respectively. No studies mentioned genotyping under blinded condition.

### Meta-analysis

**Fig 2:**
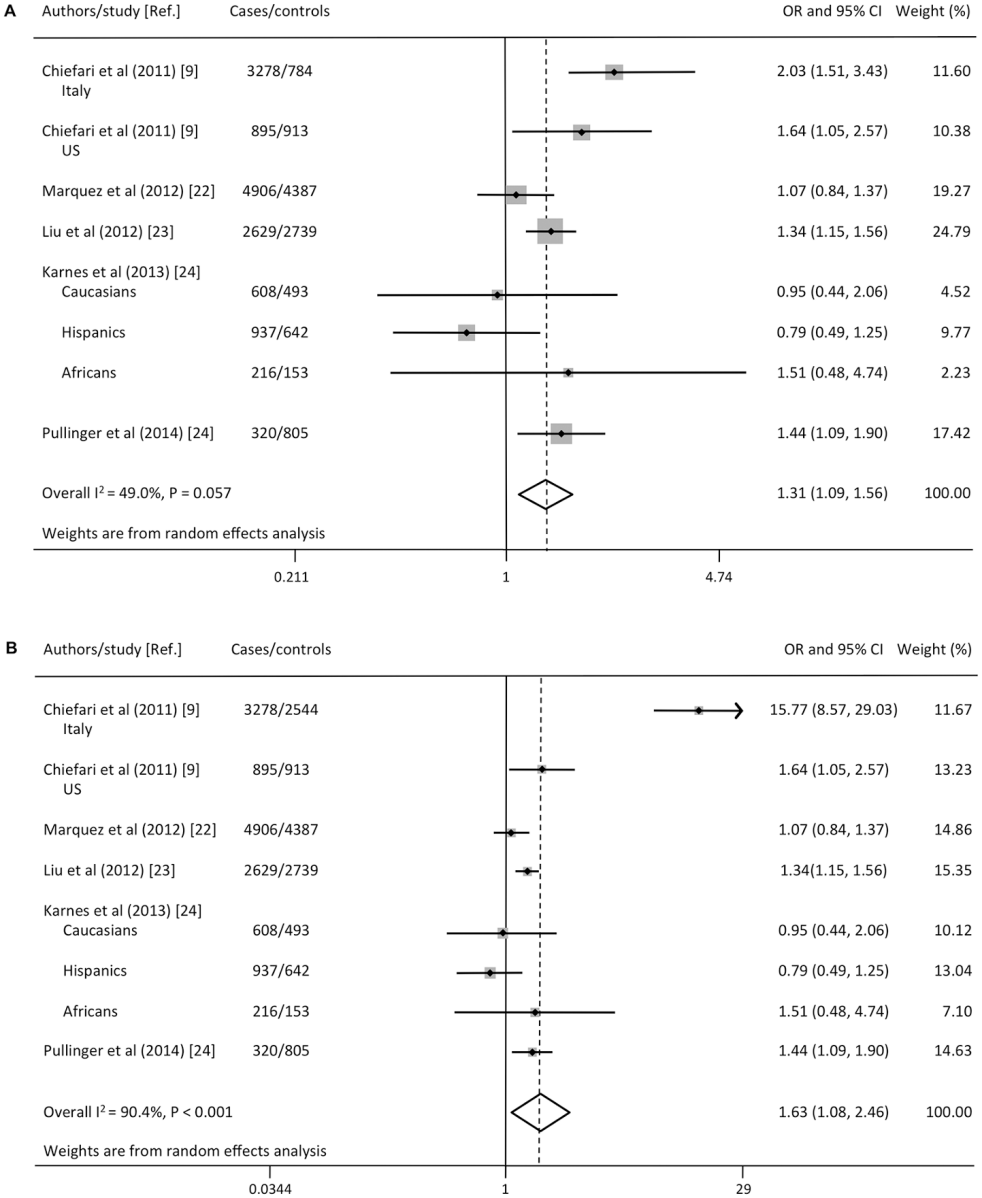
Forest plot for the relationship between *HMGA1* rs146052672 variant and T2D risk. **(A)** Meta-analysis included 784 unaffected healthy individuals, in the Italian control group, who were selected on the basis of a self-reported medical questionnaire, without a personal interview. **(B)** Meta-analysis included 2,544 interviewed healthy individuals, in the Italian control group, who had neither a personal nor a family history of T2D or related diseases.

The objective of all five selected studies was to analyze the effect of *HMGA1* rs146052672 variant on T2D. When all extracted data were pooled, 27,249 individuals were eligible for this analysis. Fig 2A shows data of meta-analysis exploring the effect of *HMGA1* rs146052672 variant on T2D risk. The combined adjusted OR estimates revealed that the rs146052672 variant genotype had an overall statistically significant effect on increasing the risk of T2D (OR 1.31; 95% CI 1.09, 1.56; P < 0.001). A moderate heterogeneity was detected in the analysis (I^2^ = 49.0%, P = 0.057). Statistical significance of the association between the *HMGA1* rs146052672 variant and T2D risk was maintained also when the largest (n = 2,544) interviewed Italian control group was included in the meta-analysis in place of the smaller (n = 784) not interviewed Italian control group (OR 1.63; 95% CI 1.08, 2.46; P = 0.02) (Fig 2B). The test for heterogeneity showed high heterogeneity among the studies (I^2^ = 90.4%, Q = 72.6, df = 7, P < 0.001).

### Sensitivity analyses

**Fig 3:**
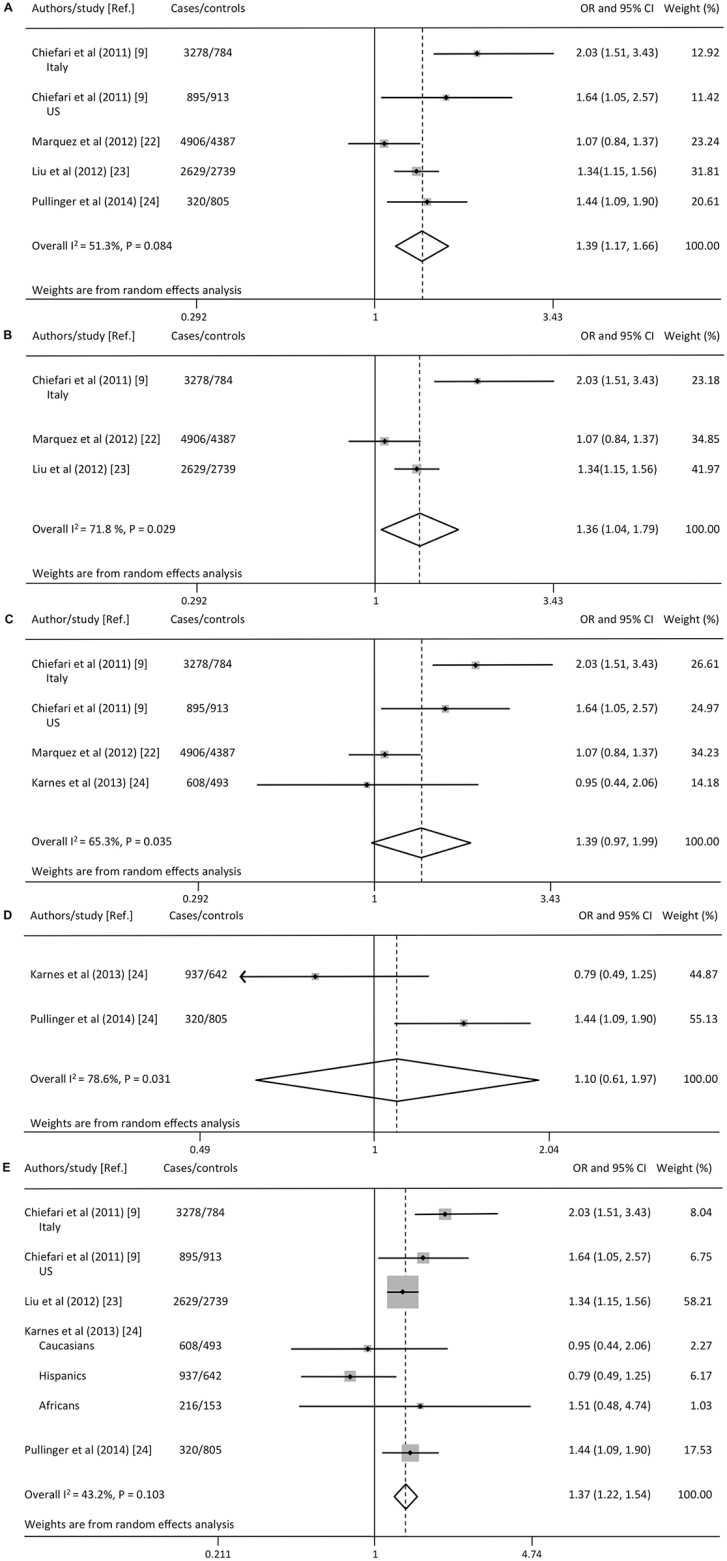
Sensitivity analysis. The effect of *HMGA1* rs146052672 variant on T2D risk was evaluated according to a set of covariates taking into account recruitment in the general population **(A)**, quality of studies (**B**), Caucasian ethnicity **(C)**, Hispanic ethnicity **(D)**, and genotyping method **(E)**.

To further evaluate the effect of *HMGA1* rs146052672 variant on T2D risk, a pooled analysis was performed for potential sources of heterogeneity by combining studies that showed similar characteristics. The pooled OR estimate for all sensitivity analyses performed did not modify substantially the conclusions of the overall meta-analysis. To determine whether modification of the inclusion criteria affected the results of this meta-analysis, we initially performed sensitivity analysis excluding the study by Karnes et al. [24], given that it was the only study performed in a specific subgroup population (hypertensive and coronary artery disease patients). The corresponding pooled OR did not remarkably change (OR 1.39; 95% CI 1.17, 1.66; P < 0.001), confirming the stability of the results (Fig 3A). A moderate heterogeneity was detected in the analysis (I^2^ = 51.3%, Q = 8.2, df = 4, P = 0.084). Meta-analysis based on high-quality studies (as determined by methodological assessment of study quality) gave a pooled OR estimate of 1.36 (95% CI 1.04, 1.79) (Fig 3B) and, therefore, without substantially modifying the conclusions of the overall meta-analysis. Homogeneity among studies was rejected as the test for heterogeneity showed I^2^ = 71.8, Q = 7.1 df = 2, P = 0.029.

In the sensitivity analysis that included studies conducted among either Caucasians (Fig 3C) or Hispanic-Americans (Fig 3D), the 95% CI included the null value (OR 1.39; 95% CI 0.97, 1.99; and OR 1.10; 95% CI 0.61, 1.97, respectively). However, in analysis of Caucasians, the P value was just above the threshold of significance (P = 0.074), and the small number of studies available for analysis may have prevented statistical significance from being obtained. The OR of 1.39 in Caucasians was similar to the OR value from the general meta-analysis (1.31), and heterogeneity was moderate (I^2^ = 65.3%, Q = 8.6, df = 3, P = 0.035) (Fig 3C). The test for heterogeneity in Hispanic-Americans metha-analysis showed I^2^ = 78.6%, Q = 4.7, df = 1, P = 0.031 (Fig 3D). Results from meta-analyses restricted to studies that used TaqMan allelic discrimination to analyze *HMGA1* mutations, showed that the rs146052672 variant genotype had an overall statistically significant effect on increasing the risk of T2D (OR 1.37; 95% CI 1.22, 1.54; P < 0.001), and low heterogeneity (I^2^ = 43.2%, Q = 10.6, df = 6, P = 0.103) after the exclusion of one study (22) for which genotyping was by HRM (Fig 3E).

### Meta-regression

**Fig 4:**
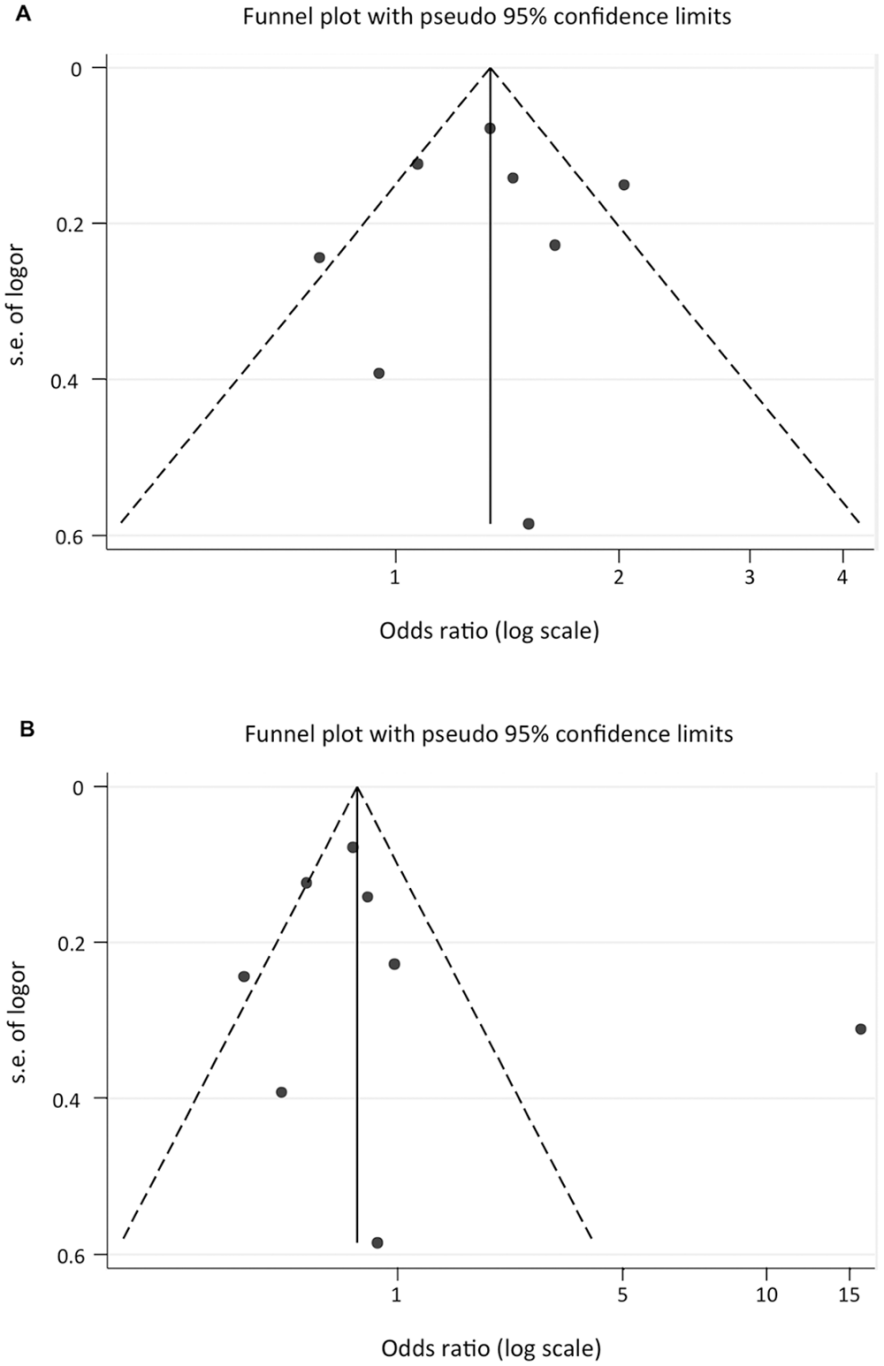
Begg’s funnel plot test of publication bias for the association of *HMGA1* rs146052672 variant and T2D risk. **(A)** Meta-analysis included 784 unaffected healthy individuals, in the Italian control group, who were selected on the basis of a self-reported medical questionnaire, without a personal interview. **(B)** Meta-analysis included 2,544 interviewed healthy individuals, in the Italian control group, who had neither a personal nor a family history of T2D or related diseases. Each round represents an individual study for the indicated association. Logor, natural logarithm of OR; s.e., standard error; perpendicular line, mean effect size.

The results of the univariate meta-regression demonstrated that none of the investigated variables affected the estimates of *HMGA1* rs146052672 variant’s effect on T2D. However, we cannot exclude a type II error due to the small number of studies. The I^2^ statistic test of homogeneity (above 90% in all meta-regression analyses) found a high heterogeneity across the various studies, and none of the characteristics investigated appeared to modify significantly the results. Funnel plots showing ORs of the individual studies *vs* the reciprocal of their standard errors did not exhibit any patent asymmetry for studies exploring the effect of *HMGA1* rs146052672 variant on T2D (Fig 4A and 4B). The P values for the Begg & Mazumdar’s adjusted rank correlation method [42], and the Egger’s regression asymmetry test [43] were P = 0.621 and P = 0.488 (Fig 4A), and P = 1.000 and P = 0.828 (Fig 4B), respectively.

## Discussion

In this meta-analysis, we evaluated the association of the rs146052672 variant in *HMGA1* gene and susceptibility to T2D, by reviewing all available published articles examining this association in different populations [9,22–25]. Our meta-analysis revealed a statistically significant increase in the risk of T2D for people with the rs146052672 variant than for those without it. Considering the resulting strength of association with the disease, it was interesting to note that the *HMGA1* rs146052672 variant represented one of the major genetic risk factor for T2D yet described, with an odds similar or even higher (OR = 1.63) than that seen with the *TCF7L2* risk alleles (OR = 1.31–1.71) [5]. A significant association of the rs146052672 variant with T2D was also confirmed when pooled analyses were conducted to explore any potential source of heterogeneity, by combining studies with high-quality scores, or that used the same genotyping method, or were performed in general populations.

The choice of choosing a suitable control group is of considerable significance and one of the most difficult aspects of case-control design [47], in which the requirement of collecting accurate and reliable data on the control group’s health status cannot be understated. To achieve comparability among cases and controls, the controls should come from the same population as the cases, they should be representative of the target population as much as possible and should be definitively unaffected [48,49]. These considerations are particularly critical especially in the case of a multifactorial and clinically heterogeneous disease such as T2D, in which a complex interplay of genetic and environmental factors may influence disease onset and progression. The importance of selecting appropriate controls in case-control studies of T2D is another interesting aspect that becomes evident in in the current meta-analysis. According to the National Diabetes Statistics Report, 2014 (www.cdc.gov/diabetes/pubs/statsreport14/national-diabetes-report%20web.pdf), more than 29 million people of the US population have diabetes. Significantly, according to the report, one in four people may not know they are diabetics. The report also indicates that 86 million Americans aged 20 years or older have prediabetes, a condition where blood glucose is higher than normal but not high enough to be diagnosed as T2D [50–52]. According to the report, 15 to 30 percent of prediabetics will develop T2D within five years, unless they make lifestyle changes (including diet and exercise). Based on these considerations, in light of the fact that many of the apparently healthy control individuals who are genetically predisposed will develop T2D later in life, it is easy to understand how difficult it can be to define an appropriate non-diabetic control group in conducting case-control studies of T2D without extensive testing and personal interviews aimed at recruiting healthy individuals with neither a personal nor family history of T2D and related conditions, including hypertension, hyperlipidemia and cardiovascular disease [9,53]. Thus, this issue could represent a misclassification bias that might lead to misleading results in studies investigating the association of T2D with certain genotypes [54], including the more recent GWAS [55–57]. Consistently with the above-mentioned remarks, the strength of association of the rs146052672 variant with T2D was significantly greater in case-control studies involving carefully screened, interviewed controls [9,23], than in studies that included healthy control individuals screened only for the absence of T2D and not having a personal interview [9].

A word of caution to the interpretation of our data is appropriate in view of the heterogeneity we found in our analysis. To address this point, we conducted sensitivity analysis and meta-regression according to characteristics retrieved from the single studies and that could have affected the overall results, such as control population recruitment, TaqMan allelic discrimination confirmed by direct sequencing as a method to analyze *HMGA1* gene mutations, and ethnicity. Although we were not able to completely rule out heterogeneity in the sensitivity analysis, this analysis allowed us to elucidate the extent to which each of the covariates investigated contributed to the overall heterogeneity, and, indeed, heterogeneity was eliminated when the method used for genotyping was involved. We are conscious that heterogeneity among ethnic groups may have impact on the results, and this is especially critical considering the disparate frequencies of the mutation among ethnic groups. However, subgroups analysis may be provided only for Caucasians and for Hispanics, as data on Asians and Africans were each from a single study. Although we were not able to reduce heterogeneity in the sensitivity analysis by ethnic groups, due to the small number of studies, we cannot exclude to have failed to find a difference. More studies in different ethnic groups are needed.

A particular strength of the present work lies in the rigorous methods used to select studies and evaluation of potential risk of bias. We did not find any evidence of publication bias in the studies reviewed here, which decreases the likelihood that our findings were due to our method of article selection. Furthermore, as this is the first meta-analysis to report an association between *HMGA1* rs146052672 variant and the risk of T2D, we perceive this as an ulterior strength of this work that should trigger further research in this direction. Conversely, as limitations of this study, we should mention that the association between *HMGA1* rs146052672 variant and T2D is not widely studied and, therefore, the number of analyzed articles was small, thus introducing a problem of inadequate statistical power, as frequently occurs in studies evaluating the role of genetic polymorphism. Nevertheless, when all extracted data were pooled, a sizeable number of subjects were eligible for analysis. Also, as this meta-analysis was based on data from case-control studies that are susceptible to selection bias, failure to consider potential confounders cannot be ruled out.

In accordance with the interim guidelines for epidemiologic credibility in the assessment of cumulative evidence on genetic associations [58], our meta-analysis may provide good evidence of the credibility of a putative association between the *HMGA1* rs146052672 variant and T2D risk. However, although an impact of the *HMGA1* rs146052672 variant on risk of T2D is supported in this meta-analysis, biological evidence for this association is still lacking; while reductions in both *HMGA1* mRNA and protein expression were reported in both blood monocytes and transformed lymphoblasts obtained from diabetic patients carrying the rs146052672 variant [9], this finding was partially challenged by examining *HMGA1* mRNA expression only in subcutaneous fat of healthy Scandinavian subjects with the variant [22], and by bioinformatic analysis on rs146052672 [24]. Though several questions have been raised and discussed about this point [59,60], there is no doubt that more investigation to explore the biological significance of the rs146052672 variant is highly desirable.

## Conclusions

This meta-analysis provides supporting evidence that the rs146052672 variant in the *HMGA1* gene might increase the risk of development of T2D. Given that the number of available published studies focusing on this issue was limited and most of the study subjects were Caucasian, further investigation is needed to see whether these findings can be generalized to other populations.

## Supporting Information

S1 Supporting Information Studies excluded from meta-analysis. (DOCX)

S2 Supporting Information Meta-analysis on genetic association studies checklist. (DOCX)

S3 Supporting Information PRISMA checklist. (DOC)
