# Supplementary material for: The Association between HMGA1 rs146052672 Variant and Type 2 Diabetes: A Transethnic Meta-Analysis
Source: PLoS One. 2015 Aug 21;10(8):e0136077. doi: 10.1371/journal.pone.0136077 (PMC4546600; doi:10.1371/journal.pone.0136077)
Supplement: S1 Supporting Information — (DOCX) [file pone.0136077.s001.docx]

**Studies excluded from meta-analysis**

**Studies excluded as inappropriate after review of title or abstract:**

1. Visscher, Peter M., et al. "Five years of GWAS discovery." *The American Journal of Human Genetics* 90.1 (2012): 7-24.
2. Simonson, Matthew A. *Polygenic analysis of genome-wide SNP data*. Diss. UNIVERSITY OF COLORADO AT BOULDER, 2013.
3. Glinskii, Anna B., et al. "Identification of intergenic trans-regulatory RNAs containing a disease-linked SNP sequence and targeting cell cycle progression/differentiation pathways in multiple common human disorders." *Cell Cycle* 8.23 (2009): 3925-3942.
4. Glinsky, Gennadi. "Disease phenocode analysis identifies SNP-guided microRNA maps (MirMaps) associated with human" master" disease genes."*Cell Cycle* 7.23 (2008): 3680-3694.
5. El-Sherbini, Sherif M., et al. "Gene polymorphism of transforming growth factor-β1 in Egyptian patients with type 2 diabetes and diabetic nephropathy." *Acta biochimica et biophysica Sinica* 45.4 (2013): 330-338.
6. Pechlivanis, Sonali, et al. "Insulin pathway related genes and risk of colorectal cancer: INSR promoter polymorphism shows a protective effect." *Endocrine-related cancer* 14.3 (2007): 733-740.
7. Veeraraghavulu, Praveen Chakravarthi, et al. "Analysis of polymorphism in Kir 6.2 gene in Type 2 diabetic patients." *Asian Journal of Biomedical and Pharmaceutical Sciences* 4.37 (2014): 15-20.
8. Zhang, Yue, et al. "The gene polymorphisms of UCP1 but not PPAR γ and TCF7L2 are associated with diabetic retinopathy in Chinese type 2 diabetes mellitus cases." *Acta ophthalmologica* (2014).
9. Zhang, Ruyi, et al. "Effects of Pro12Ala polymorphism in peroxisome proliferator-activated receptor-γ2 gene on metabolic syndrome risk: A meta-analysis." *Gene* 535.1 (2014): 79-87.
10. Fontanesi, L., et al. "Identification and association analysis of several hundred single nucleotide polymorphisms within candidate genes for back fat thickness in Italian Large White pigs using a selective genotyping approach." *Journal of animal science* 90.8 (2012): 2450-2464.
11. Schrider, Daniel R., et al. "Gene copy-number polymorphism caused by retrotransposition in humans." *PLoS genetics* 9.1 (2013): e1003242.
12. Bolormaa, Sunduimijid, et al. "A multi-trait, meta-analysis for detecting pleiotropic polymorphisms for stature, fatness and reproduction in beef cattle."*PLoS genetics* 10.3 (2014): e1004198.
13. Chiefari, Eusebio, et al. "Pseudogene-mediated posttranscriptional silencing of HMGA1 can result in insulin resistance and type 2 diabetes." *Nature communications* 1 (2010): 40.
14. Chiefari, Eusebio, et al. "HMGA1 is a novel downstream nuclear target of the insulin receptor signaling pathway." *Scientific reports* 2 (2012).
15. Iiritano, Stefania, et al. "The HMGA1-IGF-I/IGFBP system: a novel pathway for modulating glucose uptake." *Molecular Endocrinology* 26.9 (2012): 1578-1589.
16. Pink, Ryan Charles, et al. "Pseudogenes: pseudo-functional or key regulators in health and disease?." *Rna* 17.5 (2011): 792-798.
17. Pullakhandam, Raghu, et al. "Contrasting effects of type 2 and type 1 diabetes on plasma RBP4 levels: The significance of transthyretin." *IUBMB life* 64.12 (2012): 975-982.
18. Dey, Debleena, et al. "Fatty acid represses insulin receptor gene expression by impairing HMGA1 through protein kinase Cε." *Biochemical and biophysical research communications* 357.2 (2007): 474-479.
19. Brambilla, Gianfranco, and Alfredo Cantafora. "Metabolic and cardiovascular disorders in highly inbred lines for intensive pig farming: how animal welfare evaluation could improve the basic knowledge of human obesity." *Ann Ist Super Sanita* 40.2 (2004): 241-4.
20. Shepherd, Peter R., and Barbara B. Kahn. "Glucose transporters and insulin action—implications for insulin resistance and diabetes mellitus." *New England Journal of Medicine* 341.4 (1999): 248-257.
21. Polonsky, Kenneth S., Jeppe Sturis, and Graeme I. Bell. "Non-insulin-dependent diabetes mellitus—a genetically programmed failure of the beta cell to compensate for insulin resistance." *New England Journal of Medicine* 334.12 (1996): 777-783.
22. McKenney, Rachel L., and Daniel K. Short. "Tipping the balance: the pathophysiology of obesity and type 2 diabetes mellitus." *Surgical Clinics of North America* 91.6 (2011): 1139-1148.
23. Hock, Robert, et al. "HMG chromosomal proteins in development and disease."*Trends in cell biology* 17.2 (2007): 72-79.
24. Speliotes, Elizabeth K., et al. "Association analyses of 249,796 individuals reveal 18 new loci associated with body mass index." *Nature genetics* 42.11 (2010): 937-948.
25. Fezeu, L., et al. "O90 Apport des biomarqueurs de l’adiosité, de l’inflammation et de la fonction endothéliale dans la prédiction du diabète de type 2 au sein de la cohorte SU. VI. MAX." *Diabetes & Metabolism* 37.1 (2011): A22-A23.
26. Gudbjartsson, Daniel F., et al. "Many sequence variants affecting diversity of adult human height." *Nature genetics* 40.5 (2008): 609-615.
27. Zhu, Hao, et al. "The Lin28/let-7 axis regulates glucose metabolism." *Cell* 147.1 (2011): 81-94.
28. Hampton, Tracy. "Gene variant confers risk of diabetes." *JAMA* 295.9 (2006): 987-988.
29. Fan, B., et al. "Identification of genetic markers associated with residual feed intake and meat quality traits in the pig." *Meat science* 84.4 (2010): 645-650.
30. Weedon, Michael N., and Timothy M. Frayling. "Reaching new heights: insights into the genetics of human stature." *Trends in genetics* 24.12 (2008): 595-603.
31. Liu, Jimmy Z., et al. "A versatile gene-based test for genome-wide association studies." *The American Journal of Human Genetics* 87.1 (2010): 139-145.
32. Arcidiacono, Biagio, et al. "Insulin resistance and cancer risk: an overview of the pathogenetic mechanisms." *Experimental diabetes research* 2012 (2012).
33. Zeng, Zhuanping, et al. "Three single nucleotide variants of the HDAC gene are associated with type 2 diabetes mellitus in a Chinese population: A community-based case–control study." *Gene* 533.1 (2014): 427-433.
34. Lettre, Guillaume. "Genetic regulation of adult stature." *Current opinion in pediatrics* 21.4 (2009): 515-522.
35. Liu, Lei, et al. "Association between variants of EXT2 and type 2 diabetes: a replication and meta-analysis." *Human genetics* 132.2 (2013): 139-145.
36. Shepherd, Peter R., and Barbara B. Kahn. "Glucose transporters and insulin action—implications for insulin resistance and diabetes mellitus." *New England Journal of Medicine* 341.4 (1999): 248-257.
37. Bianchi, Marco E., and Alessandra Agresti. "HMG proteins: dynamic players in gene regulation and differentiation." *Current opinion in genetics & development*15.5 (2005): 496-506.
38. Capula, Carmelo, et al. "Prevalence and predictors of postpartum glucose intolerance in Italian women with gestational diabetes mellitus." *Diabetes research and clinical practice* 105.2 (2014): 223-230.
39. Herrera, Blanca M., Sarah Keildson, and Cecilia M. Lindgren. "Genetics and epigenetics of obesity." *Maturitas* 69.1 (2011): 41-49.
40. Yang, Zhenzhong, et al. "T2D@ ZJU: a knowledgebase integrating heterogeneous connections associated with type 2 diabetes mellitus." *Database*2013 (2013): bat052.
41. Allen, Hana Lango, et al. "Hundreds of variants clustered in genomic loci and biological pathways affect human height." *Nature* 467.7317 (2010): 832-838.
42. Wang, Haoran, et al. "Common Variants in KCNQ1 Confer Increased Risk of Type 2 Diabetes and Contribute to the Diabetic Epidemic in East Asians: A Replication and Meta‐Analysis." *Annals of human genetics* 77.5 (2013): 380-391.
43. Reamon-Buettner, Stella M., Si-Hyen Cho, and Juergen Borlak. "Mutations in the 3'-untranslated region of GATA4 as molecular hotspots for congenital heart disease (CHD)." *BMC medical genetics* 8.1 (2007): 38.
44. Chiefari, E., S. Tanyolaç, and F. Paonessa. "Supplementary Online Content." (2011).
45. WACHTER, KERRI. "Enterovirus Risk Is 10-Fold Greater in Type." *DIABETES*(2011).
46. dos Santos, Alessandro Brito, et al. "Basic bioinformatics tools for genotyping studies. A practical exercise with type 2 diabetes mellitus." (2013).
47. Brunetti, Antonio. "Clinical Relevance."
48. KANG, Le, et al. "Study of the plasma level of sRAGE and other related factors in first-degree relatives with normal glucose tolerance of Type 2 Diabetes."*Journal of Shandong University (Health Sciences)* 3 (2012): 002.
49. Byrne, C., and K. K. Nkongolo. "Genetic susceptibility for type 2 diabetes mellitus among North American Aboriginals." *International Journal of Medicine and Medical Sciences* 4.10 (2012): 220-231.
50. Graham, Timothy E., et al. "Retinol-binding protein 4 and insulin resistance in lean, obese, and diabetic subjects." *New England Journal of Medicine* 354.24 (2006): 2552-2563.
51. Larkina, T. A., et al. "Expression profiling of candidate genes for abdominal fat mass in domestic chicken Gallus gallus." *Russian Journal of Genetics* 47.8 (2011): 1012-1015.
52. Hertel, Jens K. Hertel, et al. "Type 2 diabetes genes–Present status and data from Norwegian studies." *Norsk epidemiologi* 23.1 (2013).
53. Chiefari, Eusebio, et al. "Transcriptional Regulation of the HMGA1 Gene by Octamer-Binding Proteins Oct-1 and Oct-2." *PloS one* 8.12 (2013): e83969.
54. Arcidiacono, Biagio, et al. "Cooperation between HMGA1, PDX-1, and MafA is Essential for Glucose-Induced Insulin Transcription in Pancreatic Beta Cells."*Frontiers in Endocrinology* 5 (2015): 237.
55. Vignini, Arianna, et al. "Alzheimer's disease and diabetes: new insights and unifying therapies." *Current diabetes reviews* 9.3 (2013): 218-227.
56. Böttcher, Yvonne, et al. "Obesity genes: implication in childhood obesity."*Paediatrics and Child Health* 22.1 (2012): 31-36.
57. Honardoost, Maryam, et al. "Insulin Resistance Associated Genes and miRNAs." *Applied biochemistry and biotechnology* 174.1 (2014): 63-80.
58. Fedele, Monica, Dario Palmieri, and Alfredo Fusco. "HMGA2: a pituitary tumour subtype-specific oncogene?." *Molecular and cellular endocrinology* 326.1 (2010): 19-24.
59. Xia, Qianghua, and Struan FA Grant. "The genetics of human obesity." *Annals of the New York Academy of Sciences* 1281.1 (2013): 178-190.
60. O'Reilly, Dawn, et al. "Differentially expressed, variant U1 snRNAs regulate gene expression in human cells." *Genome research* 23.2 (2013): 281-291.
61. Wang, Tiange, Guang Ning, and Zachary Bloomgarden. "Diabetes and cancer relationships (糖尿病与肿瘤的关联性)." *Journal of diabetes* 5.4 (2013): 378-390.
62. Foti, Daniela P., et al. "New Target Genes for the Peroxisome Proliferator-Activated Receptor-𝛾 (PPAR 𝛾) Antitumour Activity: Perspectives from the Insulin Receptor." *PPAR research* 2009 (2009).
63. Pautz, Andrea, et al. "Regulation of the expression of inducible nitric oxide synthase." *Nitric Oxide* 23.2 (2010): 75-93.
64. Lanktree, Matthew B., et al. "Meta-analysis of dense genecentric association studies reveals common and uncommon variants associated with height." *The American Journal of Human Genetics* 88.1 (2011): 6-18.
65. Semple, Robert K., et al. "Genetic syndromes of severe insulin resistance."*Endocrine reviews* 32.4 (2011): 498-514.
66. Goumidi, Louisa, et al. "Effects of established BMI-associated loci on obesity-related traits in a French representative population sample." *BMC genetics* 15.1 (2014): 62.
67. Ntzani, Evangelia E., et al. "Consistency of genome-wide associations across major ancestral groups." *Human genetics* 131.7 (2012): 1057-1071.
68. Graff, M., et al. "Generalization of adiposity genetic loci to US Hispanic women."*Nutrition & diabetes* 3.8 (2013): e85.
69. Liu, Jimmy Z., et al. "Genome-wide association study of height and body mass index in Australian twin families." *Twin Research and Human Genetics* 13.02 (2010): 179-193.
70. Qiu, Beiying, Weiping Han, and Vinay Tergaonkar. "NUCKS: a potential biomarker in cancer and metabolic disease." *Clinical Science* 128.10 (2015): 715-721.
71. Fan, B., Z-Q. Du, and M. F. Rothschild. "The Hepatocyte nuclear factor-1 alpha (HNF1A) gene is associated with fatness and loin muscle area in the pig."*animal* 4.10 (2010): 1619-1627.
72. Singh, Rajvir, et al. "LRP6 enhances glucose metabolism by promoting TCF7L2-dependent insulin receptor expression and IGF receptor stabilization in humans." *Cell metabolism* 17.2 (2013): 197-209.
73. Fumeron, Frédéric. "Genetics of the human obesities." *Physiology and Physiopathology of Adipose Tissue*. Springer Paris, 2013. 351-372.
74. Friedrichsen, Martin, et al. "Carboxylesterase 1 gene duplication and mRNA expression in adipose tissue are linked to obesity and metabolic function." *PloS one* 8.2 (2013): e56861.
75. Ramirez-Garcia, Sergio Alberto, et al. "Implicaciones en la atención primaria en salud de la genética y genómica en la diabetes mellitus tipo 2." *Rev Med Inst Mex Seguro Soc* 51.3 (2013): e6-26.
76. Hammond, Scott M., and Norman E. Sharpless. "HMGA2, microRNAs, and stem cell aging." *Cell* 135.6 (2008): 1013-1016.
77. Savona-Ventura, Charles, and Stephanie Savona-Ventura. "The inheritance of obesity." *Best Practice & Research Clinical Obstetrics & Gynaecology* (2014).
78. Kim, Kwan-Suk, et al. ". A COMPARATIVE STUDY OF OBESITY QTL AND CANDIDATE GENES IN THE PIG: A MODEL ORGANISM FOR HUMAN GENETICS AND PHYSIOLOGY." *Comparative genome analysis in the pig as a model for understanding human obesity* 1050: 38.
79. Panda, Amaresh C., et al. "Posttranscriptional regulation of insulin family ligands and receptors." *International journal of molecular sciences* 14.9 (2013): 19202-19229.
80. Badsi, Manel N., et al. "Combined effect of established BMI loci on obesity-related traits in an Algerian population sample." *BMC genetics* 15.1 (2014): 128.
81. Barter, Philip, et al. "HDL cholesterol, very low levels of LDL cholesterol, and cardiovascular events." *New England Journal of Medicine* 357.13 (2007): 1301-1310.
82. Hinney, Anke, Anna-Lena Volckmar, and Jochen Antel. "Genes and the hypothalamic control of metabolism in humans." *Best Practice & Research Clinical Endocrinology & Metabolism* 28.5 (2014): 635-647.
83. Arnoldo, Laura, et al. "A novel mechanism of post-translational modulation of HMGA functions by the histone chaperone nucleophosmin." *Scientific reports* 5 (2015).
84. Bureau, Christophe, et al. "Expression of the transcription factor Klf6 in cirrhosis, macronodules, and hepatocellular carcinoma." *Journal of gastroenterology and hepatology* 23.1 (2008): 78-86.
85. Ozturk, Nihan, et al. "HMGA proteins as modulators of chromatin structure during transcriptional activation." *Frontiers in cell and developmental biology* 2 (2014).
86. Zhang, Xiaoyang, et al. "Integrative functional genomics identifies an enhancer looping to the SOX9 gene disrupted by the 17q24. 3 prostate cancer risk locus."*Genome research* 22.8 (2012): 1437-1446.
87. Rankinen, Tuomo, et al. "The human obesity gene map: the 2005 update."*Obesity* 14.4 (2006): 529-644.
88. Malaguarnera, Roberta, and Antonino Belfiore. "The insulin receptor: a new target for cancer therapy." *Frontiers in endocrinology* 2 (2011).
89. Westley, Rosalyne L., and Felicity EB May. "A twenty-first century cancer epidemic caused by obesity: the involvement of insulin, diabetes, and insulin-like growth factors." *International journal of endocrinology* 2013 (2013).
90. Shungin, Dmitry, et al. "New genetic loci link adipose and insulin biology to body fat distribution." *Nature* 518.7538 (2015): 187-196.
91. Uth, Kristin, and Roger Sleigh. "Deregulation of the circadian clock constitutes a significant factor in tumorigenesis: a clockwork cancer. Part I: clocks and clocking machinery." *Biotechnology & Biotechnological Equipment* 28.2 (2014): 176-183.
92. del Seguro Social, I. M. S. S. "Genética de la obesidad infantil." *Rev Med Inst Mex Seguro Soc* 52.Supl 1 (2014): S78-S87.
93. Casas-Agustench, Patricia, et al. "Saturated fat intake modulates the association between an obesity genetic risk score and body mass index in two US populations." *Journal of the Academy of Nutrition and Dietetics* 114.12 (2014): 1954-1966.
94. Benton, Miles C., et al. "An analysis of DNA methylation in human adipose tissue reveals differential modification of obesity genes before and after gastric bypass and weight loss." *Genome Biology* 16.1 (2015): 8.
95. Bendall, S. C., et al. "Growth and Growth Factors." *Yearbook of Pediatric Endocrinology 2008* 448 (2008): 43.
96. Belfiore, Antonino, et al. "Insulin receptor isoforms and insulin receptor/insulin-like growth factor receptor hybrids in physiology and disease." *Endocrine reviews* 30.6 (2009): 586-623.
97. Antoshechkin, A., et al. "Analysis of effects of the herbal preparation Circulat on gene expression levels in cultured human fibroblasts." *Phytotherapy Research*21.8 (2007): 777-789.
98. Li, Jun, and Xin Lu. "The emerging roles of 3′ untranslated regions in cancer."*Cancer letters* 337.1 (2013): 22-25.
99. Seltzer, Justin, et al. "Gene and protein expression in pituitary corticotroph adenomas: a systematic review of the literature." *Neurosurgical focus* 38.2 (2015): E17.
100. Butler, Merlin G., Austen McGuire, and Ann M. Manzardo. "Clinically relevant known and candidate genes for obesity and their overlap with human infertility and reproduction." *Journal of assisted reproduction and genetics* (2015): 1-14.
101. Martins, Manoela D., and Rogerio M. Castilho. "Histones: Controlling tumor signaling circuitry." *Journal of carcinogenesis & mutagenesis* 1.Suppl 5 (2013): 1.
102. Müller, Marietta Henrike. *Dysregulation of the high mobility group AT-hook 2 (HMGA2) gene in human tumours*. Diss. University of Bremen, 2014.
103. Sharman, Mathew J., et al. "The guinea pig as a model for sporadic Alzheimer’s disease (AD): the impact of cholesterol intake on expression of AD-related genes." *PloS one* 8.6 (2013): e66235.
104. De Martino, Ivana. *Gene expression profile analysis of pituitary adenomas developed by HMGA transgenic mice*. Diss. Università degli Studi di Napoli Federico II, 2008.
105. Onteru, Suneel K., et al. "Whole genome association studies of residual feed intake and related traits in the pig." *PloS one* 8.6 (2013): e61756.
106. Mazeh, Haggi. "MicroRNA as a diagnostic tool in fine-needle aspiration biopsy of thyroid nodules." *The oncologist* 17.8 (2012): 1032-1038.
107. Stagni, Venturina, Daniela Barila, and Simonetta Santini. *Molecular Bases of Ataxia Telangiectasia: One Kinase Multiple Functions*. INTECH Open Access Publisher, 2013.
108. Sovio, U., et al. "Genetic Determinants of Height Growth Assessed Longitudinally from Infancy to." (2009).
109. Anderson, R. A., et al. "Bibliography Current World Literature Vol 15 No 4 August 2003." *Current Opinion in Pediatrics* 15 (2003): 429-442.
110. Shyh-Chang, Ng, and George Q. Daley. "Lin28: primal regulator of growth and metabolism in stem cells." *Cell Stem Cell* 12.4 (2013): 395-406.
111. Papa, Anselmo, et al. "Triple-negative breast cancer: investigating potential molecular therapeutic target." *Expert opinion on therapeutic targets* 19.1 (2015): 55-75.
112. Achille, M., et al. "Bibliography Current World Literature Vol 17 No." *Clin Oncol*2009.32 (2009): 1-8.
113. Vanichakarn, P., J. Hwa, and J. Stitham. "Cardiovascular Pharmacogenetics of Antihypertensive and Lipid-Lowering Therapies." *Current molecular medicine*14.7 (2014): 849-879.
114. Ocaña, Sonia Chávez, and Mónica Sierra Martínez. "Genética y obesidad."
115. Pei, Yu-Fang, et al. "Meta-analysis of genome-wide association data identifies novel susceptibility loci for obesity." *Human molecular genetics* 23.3 (2014): 820-830.
116. ANDRIS, Fabienne, et al. "Biologie moléculaire et cellulaire de la réponse immune."
117. Keller, Andreas, and Eckart Meese, eds. *Nucleic Acids as Molecular Diagnostics*. John Wiley & Sons, 2014.
118. Graf, Robin, et al. "Identification of LIN28B-bound mRNAs reveals features of target recognition and regulation." *RNA biology* 10.7 (2013): 1146-1159.
119. Huuskonen, Antti. "The Role of Adiposity, Growth and Inflammation Related Gene Variants in Physical Performance and Body Composition."
120. Sander, Miriam, Benjamin F. Trump, and Curtis C. Harris. "The 23rd Aspen Cancer Conference: Mechanisms of Toxicity, Carcinaogenesis, Cancer Prevention and Cancer Therapy 2008." *Molecular carcinogenesis* 48.5 (2009): 465.
121. Idowu, Michael A., et al. "Reverse engineering of drug induced DNA damage response signalling pathway reveals dual outcomes of ATM kinase inhibition."*Biodiscovery* 4 (2013).
122. Baur, J. A., et al. "Nutrition and physiological function." *Nature* 444 (2006): 337-342.
123. Pelham, Christopher James. "Novel mechanisms of vascular-specific peroxisome proliferator-activated receptor gamma in hypertension and atherosclerosis." (2012).
124. Kenny, Eimear Elizabeth. *GENOME-SCALE GENETICS*. Diss. The Rockefeller University, 2010.
125. Allen, Hana Lango, et al. "Hundreds of variants influence human height and cluster within genomic loci and biological pathways." *The role of common genetic variation in model polygenic and monogenic traits* (2010): 94.
126. Calamandrei, Gemma. "Ethological and methodological considerations in the use of newborn rodents in biomedical research." *Ann Ist Super Sanita* 40 (2004): 195-200.
127. Fetterman, Christina Dawn. *Forkhead Evolution and the FOXC1 Inhibitory Domain*. Diss. University of Alberta, 2011.
128. Wanders, Desiree. *Novel Pleiotropic Effects of Niacin*. Diss. Auburn University, 2012.
129. Bao, Bin, et al. "Metformin may function as anti-cancer agent via targeting cancer stem cells: the potential biological significance of tumor-associated miRNAs in breast and pancreatic cancers." *Annals of translational medicine* 2.6 (2014).
130. Murchison, Charles F. "The inteactions of differently expressed genes directly targeted by Wnt\[beta]-catenin signaling: implications for colorectal carcinogenesis." (2010).
131. Belda, Benjamin John. *Adipose-derived signals induce hyperplasia of the mammary epithelium in response to 10e12z conjugated linoleic acid initiated adipose dysfunction*. Diss. The Pennsylvania State University, 2009.
132. 王海震, 王莹, and 刘定干. "真核生物 mRNA 3 忆非翻译区的功能." *Progress in Biochemistry and Biophysics* 35.9 (2008): 980-985.
133. Lee, Mi Su. *The MENX syndrome: an animal model to study the role of p27 in tumor predisposition and the response of neuroendocrine tumors to therapeutic agents*. München, Technische Universität München, Diss., 2011, 2011.
134. Fu, Zhuo, Elizabeth R. Gilbert, and Dongmin Liu. "Regulation of insulin synthesis and secretion and pancreatic Beta-cell dysfunction in diabetes."*Current diabetes reviews* 9.1 (2013): 25.
135. Arora, Sarika. "Molecular Basis of Insulin Resistance and Its Relation to Metabolic Syndrome." *INSULIN RESISTANCE* (2012): 1.
136. Sparvero, Louis J., et al. "RAGE (Receptor for Advanced Glycation Endproducts), RAGE ligands, and their role in cancer and inflammation." *J Transl Med* 7.1 (2009): 17.
137. Del Blanco, Darya Gorbenko, et al. "Isolated GH deficiency: mutation screening and copy number analysis of HMGA2 and CDK6 genes." *European Journal of Endocrinology* 165.4 (2011): 537-544.
138. Schenk, Emily R., et al. "Isomerization kinetics of AT hook decapeptide solution structures." *Analytical chemistry* 86.2 (2014): 1210-1214.
139. van der Krieken, Sophie E., et al. "CCAAT/Enhancer Binding Protein in relation to ER Stress, Inflammation, and Metabolic Disturbances."
140. Uth, Kristin, and Roger Sleigh. "Deregulation of the circadian clock constitutes a significant factor in tumorigenesis: a clockwork cancer. Part I: clocks and clocking machinery." *Biotechnology & Biotechnological Equipment* 28.2 (2014): 176-183.
141. Martins, Manoela D., and Rogerio M. Castilho. "Histones: Controlling tumor signaling circuitry." *Journal of carcinogenesis & mutagenesis* 1.Suppl 5 (2013): 1.
142. Yazbek, Soha Nabil. *Analysis of genetic susceptibility to type II diabetes in mice*. Diss. Case Western Reserve University, 2010.
143. Båvner, Ann. *Molecular mechanisms of transcriptional repression by the orphan receptor SHP*. Biovetenskaper och näringslära/Biosciences and Nutrition, 2005.
144. Papa, Anselmo, et al. "Triple-negative breast cancer: investigating potential molecular therapeutic target." *Expert opinion on therapeutic targets* 19.1 (2015): 55-75.
145. Puschendorf, Mareike, et al. "2.3. The Cbx2 chromodomain and AT hook are required for heterochromatin targeting of PRC1." *Epigenetic programming of mouse pre-implantation development*: 79.
146. Carlberg, Carsten, and Ferdinand Molnár. *Mechanisms of Gene Regulation*. Springer Science & Business Media, 2013.

**Studies excluded as not case-control studies or not including rs146052672 analysis:**

1. Marquez, M., et al. "Rarely frequent variants of the HMGA1 gene could be linked to type 2 diabetes." *DIABETES & METABOLISM*. Vol. 37. 21 STREET CAMILLE DESMOULINS, ISSY, 92789 MOULINEAUX CEDEX 9, FRANCE: MASSON EDITEUR, 2011.
2. Marquez, Marcel. *Optimisation de la technique de BRET: étude de l’association des variants d’HMGA1 avec le diabète de type 2*. Diss. Lille 2, 2011.
3. Chieffari, E., et al. "O87 Les variants fonctionnels d’HMGA1 sont fréquents et exposent à un haut risque de diabète de type 2." *Diabetes & Metabolism* 37.1 (2011): A22.
4. Garg, Abhimanyu. "HMGA1, a novel locus for type 2 diabetes mellitus." *JAMA*305.9 (2011): 938-939.
5. Froguel, Philippe, Marcel Marquez, and Stéphane Cauchi. "Response to comment on: Marquez et al. Low-frequency variants in HMGA1 are not associated with type 2 diabetes risk. Diabetes 2012; 61: 524–530." *Diabetes*61.9 (2012): e15.
6. Brunetti, Antonio, et al. "Comment on: Marquez et al. Low-Frequency Variants in HMGA1 Are Not Associated With Type 2 Diabetes Risk. Diabetes 2012; 61: 524–530." *Diabetes* 61.5 (2012): e3-e3.
7. Riu Pastor, Efrén. "Study of the role of the overexpression of the high mobility group-AT-hook-1 (HMGA1) protein in the adipose tissue and its implications in the development of obesity and insulin resistance."
8. Brunetti, Antonio, Eusebio Chiefari, and Daniela Foti. "Il contributo della genetica alla patogenesi del diabete mellito di tipo 2." *Recenti Prog Med* 102 (2011): 468-475.
9. Brunetti, Antonio, Eusebio Chiefari, and Daniela Foti. "Recent advances in the molecular genetics of type 2 diabetes mellitus." *World journal of diabetes* 5.2 (2014): 128.
10. Brunetti, A., E. Chiefari, and D. Foti. "[Perspectives on the contribution of genetics to the pathogenesis of type 2 diabetes mellitus]." *Recenti progressi in medicina* 102.12 (2011): 468-475.
11. Sakai, Kensuke, et al. "Replication study for the association of 9 East Asian GWAS-derived loci with susceptibility to type 2 diabetes in a Japanese population." *PloS one* 8.9 (2013): e76317.

**Studies excluded as partially duplicate study:**

1. Chiefari, Eusebio, et al. "A polymorphism of HMGA1 is associated with increased risk of metabolic syndrome and related components." *Scientific reports* 3 (2013).
